# Multimorbidity prevalence and patterns in chronic kidney disease: findings from an observational multicentre UK cohort study

**DOI:** 10.1007/s11255-023-03516-1

**Published:** 2023-02-21

**Authors:** Grace Hawthorne, Courtney J. Lightfoot, Alice C. Smith, Kamlesh Khunti, Thomas J. Wilkinson

**Affiliations:** 1 NIHR Applied Research Collaboration (ARC) East Midlands, Leicester Diabetes Centre, Leicester General Hospital, University of Leicester, Gwendolen Road, Leicester, LE5 4PW UK; 2 Leicester Kidney Lifestyle Team, Department of Health Sciences, University of Leicester, Leicester, UK

**Keywords:** Chronic kidney disease, Multimorbidity, Comorbidity, Transplant

## Abstract

**Purpose:**

Multimorbidity [defined as two or more long-term conditions (LTCs)] contributes to increased treatment and medication burden, poor health-related quality of life, and worse outcomes. Management strategies need to be patient centred and tailored depending on existing comorbidities; however, little is known about the prevalence and patterns of comorbidities in people with chronic kidney disease (CKD). We investigated the prevalence of multimorbidity and comorbidity patterns across all CKD stages.

**Methods:**

Multimorbidity was assessed, using a composite of self-report and clinical data, across four CKD groups stratified by eGFR [stage 1–2, stage 3a&b, stage 4–5, and kidney transplant (KTx)]. Principal component analysis using varimax rotation was used to identify comorbidity clusters across each group.

**Results:**

Of the 978 participants (mean 66.3 ± 14 years, 60% male), 96.0% had multimorbidity. In addition to CKD, the mean number of comorbidities was 3.0 ± 1.7. Complex multimorbidity (i.e. ≥ 4 multiple LTCs) was identified in 560 (57.3%) participants. When stratified by CKD stage, the two most prevalent comorbidities across all stages were hypertension (> 55%) and musculoskeletal disorders (> 40%). The next most prevalent comorbidity for CKD stages 1–2 was lung conditions and for CKD stages 3 and 4–5 it was heart problems. CKD stages 1–2 showed different comorbidity patterns and clustering compared to other CKD stages.

**Conclusion:**

Most people across the spectrum of CKD have multimorbidity. Different patterns of multimorbidity exist at different stages of CKD, and as such, clinicians should consider patient comorbidities to integrate care and provide effective treatment strategies.

**Supplementary Information:**

The online version contains supplementary material available at 10.1007/s11255-023-03516-1.

## Introduction

The prevalence of chronic kidney disease (CKD) increases with age, and is often part of a presentation of multiple long term conditions (MLTCs) [1, 2]. The presence of two or more long-term conditions (LTCs), also termed multimorbidity [3], is a well-established predictor of worse clinical outcomes and higher healthcare costs [4, 5]. Comorbidities (any additional chronic condition in association with an index condition of interest, e.g. CKD) add to overall disease and treatment burden, disease progression, transplantation rejection, medication management, quality of life (QoL), and survival [2, 6–10]. A large proportion of people living with CKD are burdened with symptoms of depression and anxiety, regardless of disease stage [11, 12], and furthermore, elevated symptoms of anxiety can increase the risk of comorbidities [12]. Hospitalisation rates in people with CKD are 2–3 times higher in those with multimorbidity [13] and additional comorbidities may lead to reductions in one’s ability to cope and self-manage their condition [14].

Clinical guidelines are often ‘disease-centred’ rather than ‘person-centred’; however, chronic conditions rarely occur in isolation and guidelines do not consider the potential for drug interactions or competing therapeutic objectives [15]. Many existing management strategies are directed toward single conditions and the global management of multimorbidity is a challenge to healthcare systems [16]. In nephrology, where multimorbidity prevalence is high [1, 2, 8], it is essential to optimise clinical care for people with comorbidities by reducing treatment burden, such as multiple appointments or polypharmacy [1, 17]. Understanding which comorbidities are common at different stages of CKD would help prevention and management strategies. This knowledge may help to identify optimal person-orientated care, tailor or integrate treatments, reduce healthcare service use, and focus resources effectively [7, 16].

According to the National Institute for Health and Care Excellence (NICE), knowledge of multimorbidity and comorbidity patterns may provide essential information for developing guidelines that offer clinical management and treatment decision support [3]. In kidney research, most existing studies have considered the epidemiological ‘combinations’ between CKD and single comorbid conditions, often through frequency counts. As guidelines are unlikely to cover all combinations of conditions, an important step would be to identify common groupings or ‘clusters’ of comorbidities that could form the basis of a simplified integrated care model [7, 18]. The use of cluster-based analysis has been applied to multimorbidity research in a range of clinical and adult populations [7, 19], although, to our knowledge, no research to date has investigated clustering patterns of comorbidities in people with CKD. Furthermore, previous multimorbidity research has focused on those predominantly with advanced CKD [1, 2, 20, 21] and there remains limited investigation post-transplant or in earlier CKD stages, where the majority of patients are managed. The aims of this study were to: (i) explore the prevalence of multimorbidity in people with CKD, and (ii) investigate how multimorbidity patterns differ across all disease stages.

## Materials and methods

### Recruitment, study design and participants

Data was collected as part of a multicentre observational cross-sectional study (DIMENSION-KD, ISRCTN84422148). The study was granted ethical approval by the Leicester Research Ethics Committee (18/EM/0117). All patients provided informed written consent and the study was conducted in accordance with the Declaration of Helsinki.

Participants were recruited from general nephrology clinics or from GP practices, between July 2018 and February 2020. 14 primary and secondary care sites in England, UK, recruited from general nephrology clinics, whilst one site (University Hospitals of Leicester NHS Trust) also recruited from eight local GP practices. Participants completed a self-administered survey pack made up of different lifestyle and health-related QoL questionnaires.

At primary care sites, eligible patients with CKD were identified by either the GP or practice manager. Eligible participants were then sent a consent form and survey pack in the post along with a stamped addressed return envelope.

At secondary care sites, eligible patients identified by the nephrologist were approached in general nephrology outpatient clinics by research staff and asked if they would like to take part. Eligible participants completed the consent form and the survey in the clinic. Participants had the option of taking the documents home and sending back later.

Participants were included if they: (1) had a confirmed clinical diagnosis of CKD from clinical data in medical records (CKD stages 1–5); (2) were aged 18 years or older; and (3) were able to provide informed consent. Participants were excluded if they were under the age of 18 years old, undergoing dialysis treatment as a form of renal replacement therapy or unable to provide written consent.

### Demographic and health variables

Basic demographic (i.e., age, sex, ethnicity) and health-related variables (e.g., smoking status) were self-reported using the survey pack. Upon receipt of the survey, participant’s clinical data, including renal function (EPI-CKD derived estimated glomerular filtration rate [eGFR] [22]), hemoglobin (Hb), albumin, albumin:creatinine ratio (ACR), blood pressure (BP), and body mass index (BMI) were extracted, where possible, from medical records. Only measurements recorded within 4 weeks prior to survey completion were included.

### Multimorbidity

Multimorbidity was defined as the presence of two or more LTCs [3]. We defined the presence of four or more LTCs as ‘complex multimorbidity’ [23]. In this analysis, this *included* CKD. Comorbidities were defined as the presence of any other LTCs, *in addition* to participants’ CKD (i.e. the index disease).

Self-reported comorbidity counts were assessed based on a composite of participant self-report or derived from clinical data (e.g., BP, BMI). Self-reported conditions were cross-referenced against those documented in their medical records where possible. The eleven comorbidities included: hypertension, obesity, diabetes (type 1 or 2), heart disease (coronary artery disease, ischaemic heart disease, atrial fibrillation, or other cardiac arrhythmias), cerebrovascular disease (stroke), peripheral vascular disease or circulatory problems, chronic respiratory disorder, cancer, depression and/or anxiety (or other mental health problems), liver disease, and any musculoskeletal (MSK) conditions. These comorbidities were chosen for pragmatic reasons including ease of identification from participant report and clinical data, and because they represent a broad spectrum of chronic conditions prevalent among people with CKD.

Hypertension was confirmed through assessment of uncontrolled blood pressure taken from the medical notes and defined as systolic BP ≥ 130 mmHg *or* diastolic BP ≥ 85 mmHg or the use of anti-hypertensive medication. Obesity was defined as a BMI of > 30 kg/m^2^. Chronic respiratory disorders were defined as the presence of asthma, chronic obstructive pulmonary disease (COPD), emphysema, or chronic bronchitis. Musculoskeletal conditions included arthralgia (joint pain) and muscle, joint (osteoarthritis) or bone (osteoporosis) problems.

### Statistical analysis

Participants were divided into four groups according to their CKD stage or whether they had received a kidney transplant. CKD stages were stratified by eGFR values obtained from participants’ medical records: stage 1–2, 60–90 mL/min/1.73 m^2^; stage 3a&b, 30–59 mL/min/1.73 m^2^; and stage 4–5, < 30 mL/min/1.73 m^2^. The transplant group (KTx) was anyone identified as a kidney transplant recipient.

In total, 1056 participants returned survey packs to the research team. 78 participants did not provide comorbidity data and were excluded from the analysis. These participants had similar demographic characteristics as the remaining 978 participants that were analysed. Of these, 283 did not have an eGFR measurement and were not identified as being a kidney transplant recipient so they could not be classified into a CKD stage; however, they were included in analysis of the total cohort. A STROBE diagram can be found in Supplementary Figure S1.

Data were analysed and graphs were generated using IBM SPSS Statistics (v28.0.1) and GraphPad Prism (v9.0.0). Continuous variables distributions were assessed for normality. Data are reported as mean ± standard deviation (SD) or median (inter-quartile range) and differences between groups were assessed using ANOVA. Frequency comparisons between groups were assessed using the Fisher test, with *α* = 0.05. To investigate comorbidity differences between groups, ANCOVA was used for continuous variables and binary logistic regression for categorical variables, adjusted for age and sex (Table 2). Comparisons were not adjusted for ethnicity as the majority (91.8%) were White British. Associations between the number of LTCs and age and sex were analysed using logistic regression. The factorability of the data were evaluated against a Kaiser–Meyer–Olkin (KMO) value > 0.60 and the significance of Bartlett’s test of Sphericity [24]. To identify comorbidity clusters, we conducted principal component analyses (PCA) by stages. We applied orthogonal (varimax) rotation to account for potential correlation between clusters. For determining the optimal number of clusters, we considered the following: Eigenvalues (> 1); scree plots; clarity of clustering patterns (i.e. variables loading strongly onto one cluster only, no clusters of single variables); and the variance explained by each cluster. We assigned comorbidities to the cluster for which they had the highest factor loading, but only if this loading was > 0.5 [25] and was > 0.2 higher than loadings for other clusters [26].

## Results

### Participant characteristics

Table 1 shows the descriptive and clinical characteristics of participants included, in total and by group. Overall, 60.0% were male, the mean age was 66.3 ± 14.0 years, and eGFR was 36.6 ± 21.0 mL/min/1.73 m^2^. There were significant effects for age, eGFR, albumin, haemoglobin, ACR, and smoking status across stages 1–5. BMI was also significantly different between all groups (Table 1).

**Table 1:** Participant and clinical characteristics of the total study population, and by stage of CKD

	Total	Stage 1–2	Stage 3a&b	Stage 4–5	Transplant	*P* value^1^	*P* value^2^
Number (%)	978	56 (5.7)	185 (18.9)	278 (28.4)	176 (18.0)		
Age (years)^a^	66.3 ± 14.0	59.1 ± 16.7	64.9 ± 12.2	69.0 ± 13.8	59.5 ± 12.9	< 0.001	< 0.001
Male, *n* (%)	587 (60.0)	29 (51.8)	117 (63.2)	181 (65.1)	101 (57.4)	0.216	0.186
Ethnicity, *n* (%)^b^							
White British	898 (91.8)	52 (96.3)	167 (92.7)	259 (93.5)	153 (86.9)	0.558	0.159
South Asian	23 (2.4)	1 (1.8)	7 (3.8)	3 (1.1)	8 (4.5)	0.120	0.082
Other white	29 (3.0)	1 (1.8)	5 (2.8)	10 (3.6)	8 (4.5)	0.814	0.762
Other	17 (1.7)	0	1 (0.6)	5 (1.8)	7 (4.0)	0.480	0.104
Clinical characteristics							
eGFR (mL/min/1.73 m^2^)^c^	36.6 ± 21.0	74.1 ± 12.2	42.6 ± 8.6	18.3 ± 6.1	48.3 ± 20.2	< 0.001	< 0.001
BMI^d^	28.6 ± 6.3	28.5 ± 7.2	29.2 ± 5.8	28.6 ± 6.5	26.6 ± 5.4	0.411	0.002
Albumin (g/L)^e^	40.6 ± 4.5	40.3 ± 5.3	41.6 ± 4.2	39.8 ± 4.6	41.2 ± 3.8	< 0.001	< 0.001
Haemoglobin (g/L)^f^	121.6 ± 22.3	132.3 ± 23.9	128.1 ± 19.7	115.0 ± 22.5	121.3 ± 21.3	< 0.001	< 0.001
ACR (mg/g)^g^	126.9 ± 197.4	201.6 ± 242.6	88.8 ± 118.9	198.1 ± 256.3	82.2 ± 162.1	0.006	< 0.001
Smoking status, *n* (%)^h^							
Smoker	66 (7.0)	7 (13.0)	15 (8.6)	13 (4.8)	9 (5.2)	0.040	0.084
Never smoked	408 (43.2)	21 (38.9)	82 (47.1)	107 (39.3)	86 (50.0)	0.268	0.103
Ex-smoker	470 (49.8)	26 (48.1)	77 (44.3)	152 (55.9)	77 (44.8)	0.044	0.048

Data are presented as mean ± SD unless otherwise stated. *P* value^1^ compares non-dialysis CKD stages 1–5, and *P* value^2^ compares all stages (i.e. including those with a transplant)

*ACR* albumin to creatinine ratio, *BMI* body mass index, *eGFR* estimated glomerular filtration rate

^a^Missing data (*n* = 6, 0.6%)

^b^Missing data (*n* = 11, 1.1%)

^c^Missing data (*n* = 297, 30.4%)

^d^Missing data (*n* = 171, 17.5%)

^e^Missing data (*n* = 308, 31.5%)

^f^Missing data (*n* = 285, 29.1%)

^g^Missing data (*n* = 716, 73.2%)

^h^Missing data (*n* = 34, 3.5%)

### The prevalence of multimorbidity and comorbidities

936 (96.0%) of the total population were multimorbid, with only 4% (*n* = 42) having CKD and *no other condition*. Of all 978 participants, in addition to CKD, 149 participants (15.2%) presented with one comorbidity, 227 participants (23.2%) had two comorbidities, and 205 participants (21.0%) had three comorbidities. As well as CKD, between 7.1 and 9.0% of participants had six or more comorbidities. The mean number of comorbidities (in addition to existing CKD) for all participants was 3.0 ± 1.7, and this was positively associated with increasing age (estimate, 95% CI, *p* value 0.028, 0.040–0.017, *p* < 0.001). 560 participants (57.3%) had complex multimorbidity, and compared to other groups, KTx had significantly lower complex multimorbidity (Table 2). Age was significantly different between those who were multimorbid and those who had CKD only (57.1 ± 16.1 vs 66.7 ± 13.8 years, *p* < 0.001, see Supplementary Table S1).

**Table 2:** Prevalence of comorbidities in the total study population, and by stage of CKD

	Total (*n* = 978)	Stage 1–2 (*n* = 56)	Stage 3a&b (*n* = 185)	Stage 4–5 (*n* = 278)	Transplant (*n* = 176)	*P* value^1^	*P* value^2^
CKD only, *n* (%)	42 (4.0)	1 (1.8)	8 (4.3)	7 (2.5)	14 (8.0)	0.321	0.168
Multimorbidity, *n* (%)	936 (96.0)	55 (98.2)	177 (95.7)	271 (97.5)	162 (92.0)	0.321	0.168
Complex MM, *n* (%)	560 (57.3)	30 (53.6)	113 (61.1)	184 (66.2)	76 (43.2)	0.529	0.006
No. of comorbidities	3.0 ± 1.7	2.9 ± 1.5	3.0 ± 1.7	3.3 ± 1.6	2.7 ± 1.8	0.561	0.144
Total no. of comorbidities, *n* (%)							
1	149 (15.2)	9 (16.1)	24 (13.0)	31 (11.2)	35 (19.9)	0.725	0.412
2	227 (23.2)	16 (28.6)	40 (21.6)	56 (20.1)	51 (29.0)	0.547	0.258
3	205 (21.0)	12 (21.4)	46 (24.9)	69 (24.8)	27 (15.3)	0.967	0.212
4	173 (17.7)	12 (21.4)	38 (20.5)	53 (19.1)	20 (11.4)	0.860	0.208
5	93 (9.5)	2 (3.6)	13 (7.0)	38 (13.7)	14 (8.0)	0.058	0.132
≥ 6	89 (9.0)	4 (7.1)	16 (8.6)	24 (8.6)	15 (8.5)	0.961	0.987

Data are presented as mean ± SD unless otherwise stated. *P* value^1^ compares non-dialysis CKD stages 1–5, and *P* value^2^ compares all stages (i.e. including those with a transplant), both adjusted for age and sex

*CKD* chronic kidney disease, *MM* multimorbidity

**Fig. 1:**
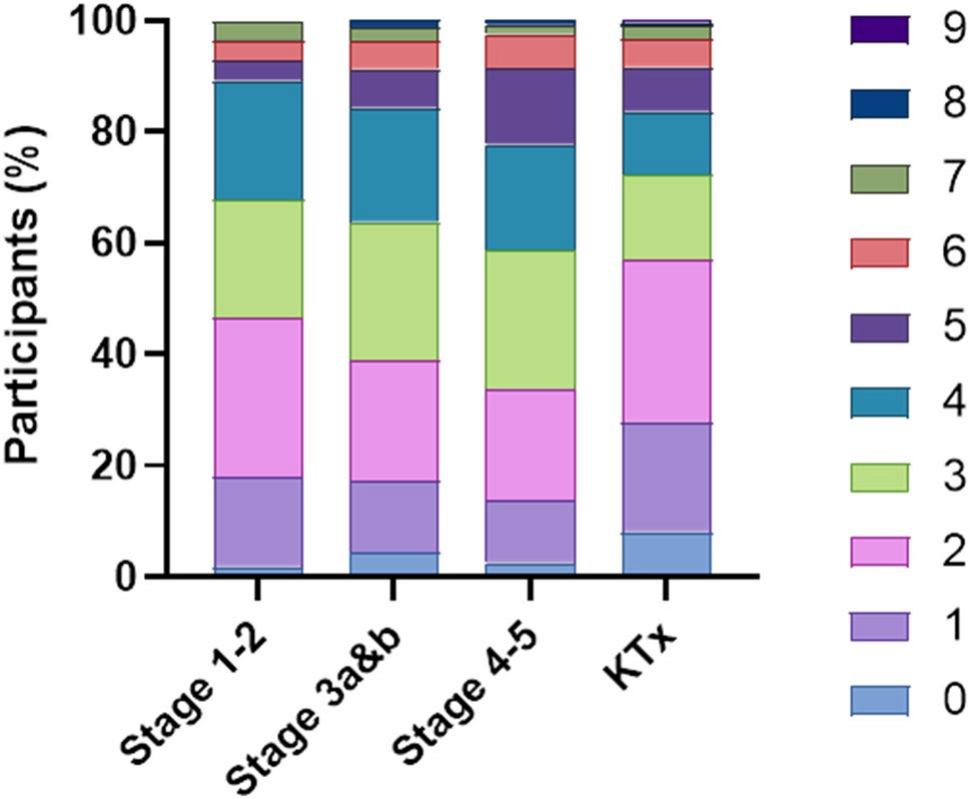
Distribution of the total number of comorbidities at each stage of CKD. Zero (0) represents no additional comorbidities to the existing CKD in the population. KTx: Kidney transplant

The prevalence of multimorbidity and the number of comorbidities was consistent across stages (Table 2). Figure 1 shows changes in frequency of comorbidities as CKD progresses. While the presence of five comorbidities approximately doubles from stages 1–2 to stage 3 and to stages 4–5 (3.6% vs 7.0% vs 13.7%), these were not significantly different when adjusted for age and sex (*p* = 0.058). In stages 1–2, the total number of comorbidities could not be predicted by age (*p* = 0.096). For stage 3, stages 4–5, and the KTx group, an increase in age was significantly associated with a greater number of comorbidities (*p* < 0.001, *p* = 0.015 and *p* = 0.003, respectively, see Supplementary Table S2).

**Fig. 2:**
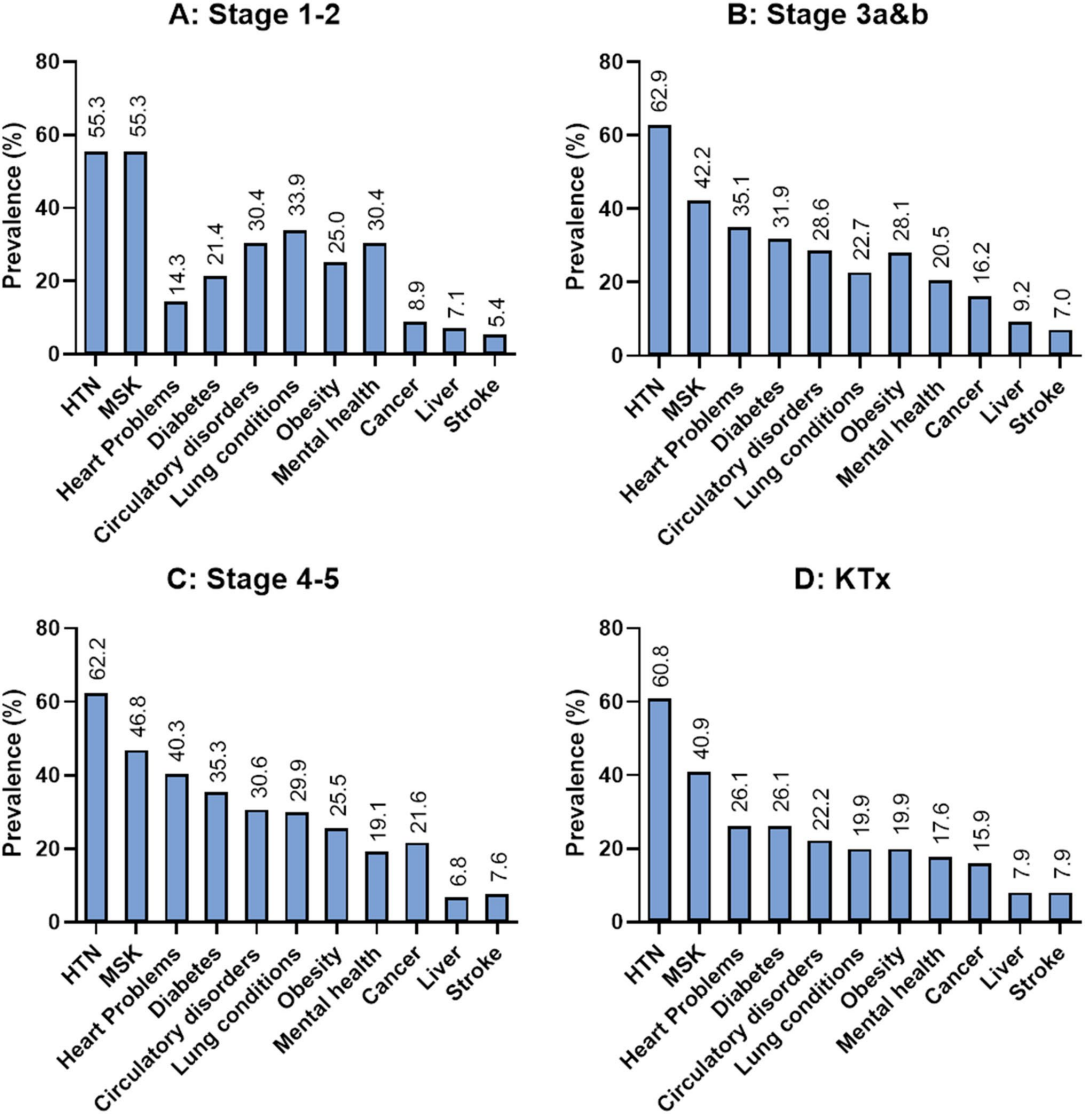
Prevalence of comorbidities for; **A** Stages 1–2; **B** Stages 3a&b; **C** Stages 4–5; **D** KTx. Comorbidities presented in order of frequency of comorbidities in the total study population (data not shown). *HTN* hypertension, *KTx* Kidney transplant, *MSK* musculoskeletal disorders

For the total population, the most prevalent comorbidities were hypertension (50.3%), MSK disorders (45.7%), and heart problems (35.8%) (Supplementary Table S3). When stratified by group, the two most prevalent comorbidities across all stages were hypertension (> 55%) and MSK disorders (> 40%). For stages 1–2, the third most prevalent comorbidity was lung conditions (33.9%, Fig. 2A). For stages 3 and 4–5, and KTx, the third most prevalent comorbidity was heart problems (35.1%, 40.3%, and 26.1% respectively, Fig. 2B, C and D).

The prevalence of heart problems was significantly different across the three stages (1–2, 3, 4–5) when adjusted for age and sex, and this increased by threefold with disease progression (14.3% vs 35.1% vs 40.3%, *p* < 0.001, Fig. 2). Although not statistically significant when adjusted for age and sex, cancer was shown to be more prevalent in the advanced stages of CKD (8.9% to 16.2% to 21.6%, *p* = 0.342).

### Patterns of multimorbidity

**Fig. 3:**
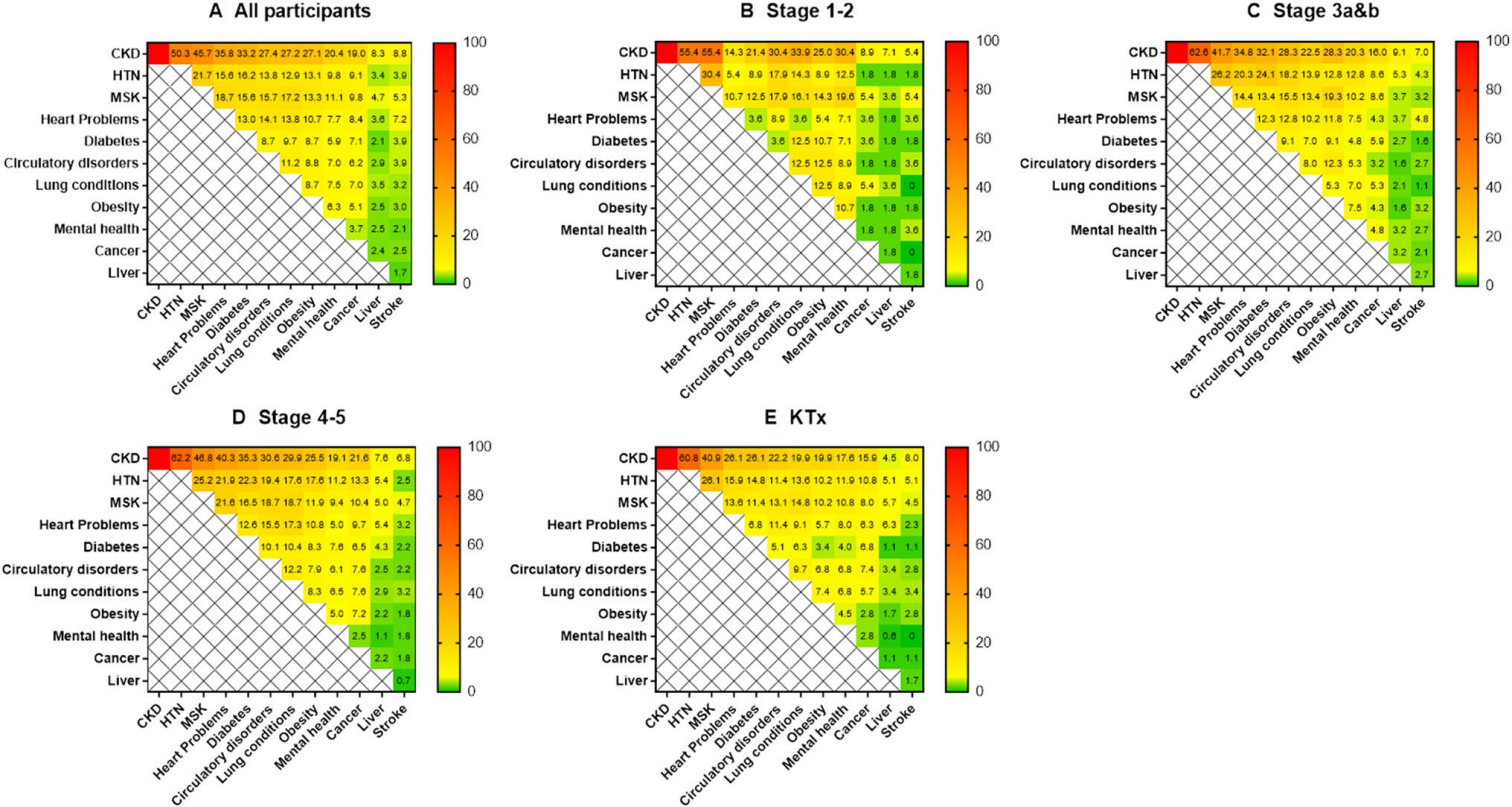
Patterns of comorbidity pairs for all participants and each stage of CKD. *KTx* Kidney transplant

For the total population and across all stages, the highest co-prevalent comorbidities were hypertension and MSK disorders. For stages 1–2 the next most common comorbidity pairing was mental health and MSK disorders (19.6%), and for both stages 3 and 4–5, the next most prevalent comorbidity pairing was diabetes and hypertension (24.1% and 22.3%, respectively) (Fig. 3).

**Fig. 4:**
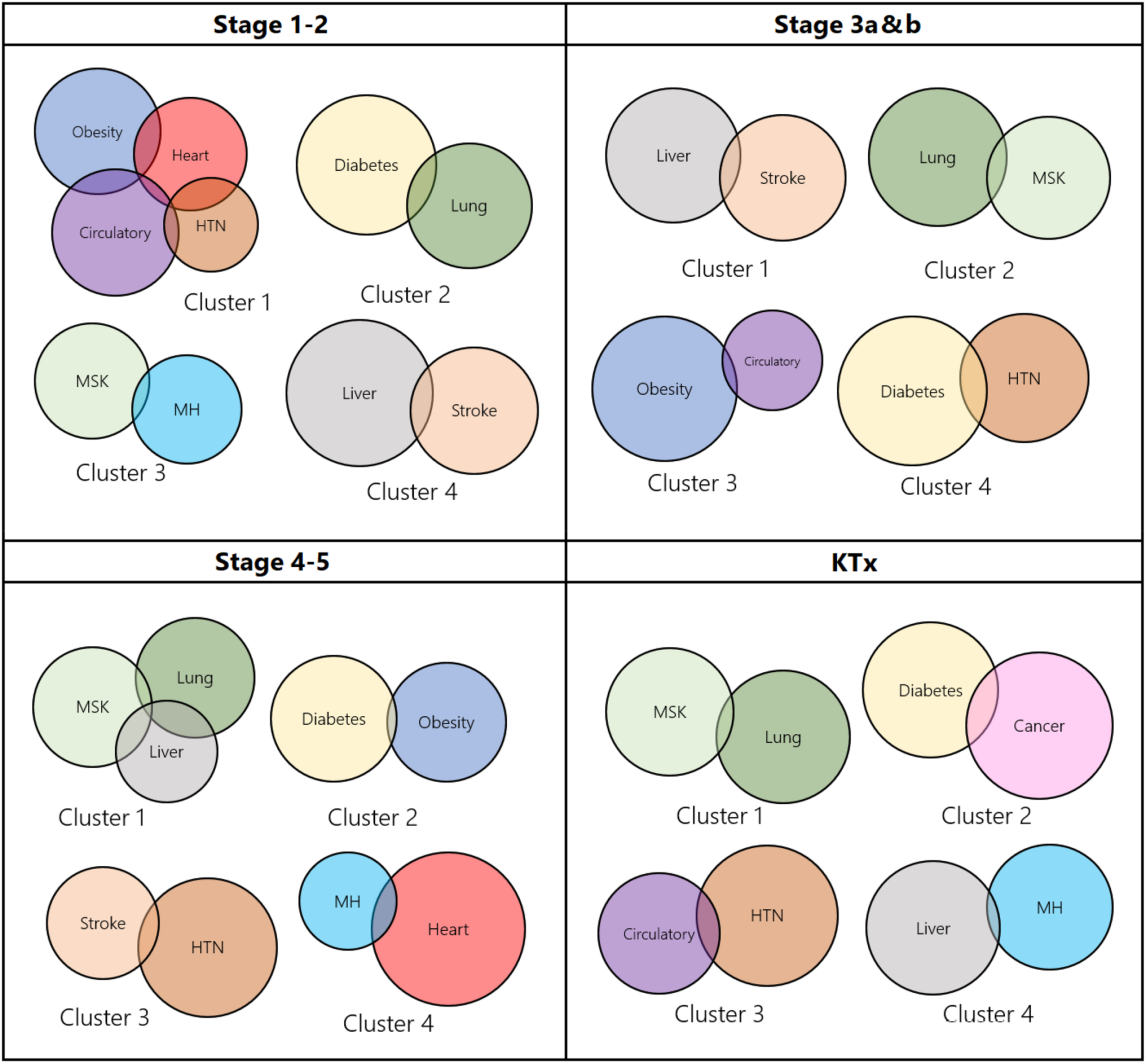
Visualisation of comorbidity clusters for each stage of CKD. Data used to make this figure are in Supplementary Table S4. This table indicates the factor loadings following PCA, and coefficients are used to denote the size of the circles. Supplementary Table S5 shows the comorbidity clusters. For stages 1–2, cancer did not cluster with any other comorbidities. For stages 3a and b, mental health disorders, heart problems, and cancer did not cluster with any other comorbidities. For stages 4–5, circulatory disorders and cancer did not cluster with any other comorbidities. For KTx, heart problems, stroke and obesity did not cluster with any other comorbidities. *HTN* hypertension, *KTx* Kidney transplant, *MH* mental health disorders, *MSK* musculoskeletal disorders

Figure 4 shows the comorbidity clusters. Comorbidity clusters appearing more than once include; liver conditions and stroke; hypertension and circulatory disorders; obesity and circulatory disorders; and lung and MSK disorders.

## Discussion

Research into multimorbidity is growing in CKD with people having to manage their CKD and concurrent MLTCs. However, much of the current research is focused on advanced CKD with little attention given to the multimorbidity burden of earlier stages of the disease (e.g. stages 1–2), post-transplant, or those that may predominantly be treated in primary care. In this study, we found that, among people living with CKD, multimorbidity is highly prevalent regardless of stage, although patterns of comorbidities appear to be different with disease progression. Hypertension and MSK disorders are the most prevalent comorbidities across all stages. Beyond these, cardiometabolic conditions such as diabetes, obesity and heart problems appear more prevalent with advancing CKD, and lung conditions and mental health disorders most prevalent in those with stages 1–2. As such, all stages of CKD are burdened with multimorbidity and as CKD progresses, people may require specialised care for, and management of, different comorbidity clusters.

Our findings are in line with previous studies that suggest multimorbidity is common among CKD populations [1, 20, 21]. In the present study, 96% of the total CKD population were multimorbid, similar to that reported by Fraser et al. [1] in a CKD stage 3 cohort of ~ 1700 participants and to the 92.6% reported in a Japanese population (~ 4500 participants) with CKD stages 1–5 [21]. In accordance with previous studies that have examined the more advanced stages (3–5) [1, 2, 13], we too identified multimorbidity in the majority of people with stages 3 and 4–5. However, in contrast to Tanaka et al. [21] who reported significantly lower multimorbidity in a younger stages 1–2 cohort (78.3%) compared to other stage subgroups (≥ 92.0%), we found that multimorbidity was also highly prevalent even at stages 1–2 (98.2%) and this was comparable to other stages. Complex multimorbidity (i.e. ≥ 4 MLTCs) was identified in over half of all participants. While it is known that advanced CKD is associated with high disease and treatment burden [1, 8, 17], these data suggest that even people living with mild CKD are burdened with multimorbidity. As such, people with early CKD may already struggle to self-manage multiple conditions, not engage successfully with healthcare services, and be burdened with a complex treatment regime and poor QoL.

We observed more complex multimorbidity as CKD progressed; however, the mean number of comorbidities was not significantly different. Evidence suggests that, in a CKD population, the number of comorbidities is influenced by age [1] and we found similar observations in our analysis. While differences in the prevalence of multimorbidity and comorbidities in CKD between studies may be owing to differences in age, this does not refute the fact that people with advancing CKD suffer with more comorbidities and need to be managed accordingly.

Given its role in disease pathophysiology, hypertension is frequently reported as a common concordant comorbidity (i.e. share pathophysiologic profile) in CKD [1, 2, 21, 27] and unsurprisingly we identified hypertension as the most prevalent comorbidity across all stages (> 55% of participants). In contrast to some previous studies, MSK disorders (> 40% of participants) was the second most prevalent comorbidity in all stages. Although some studies have reported a low prevalence of specific MSK disorders (< 10%) [2, 21], MSK-related pain is a common symptom among those with CKD [27, 28]. Our findings support other studies that observed a moderate prevalence of chronic MSK pain (53.3%) and MSK disorders (58.6%) in a CKD population [29, 30]. Discrepancies in the literature may be owing to the characterisation of comorbidities, as chronic pain, which will frequently incorporate MSK pain, is often classified as a comorbidity [1, 2, 31]. MSK-related comorbidities are often recognised as discordant comorbidities that could contribute to poor patient outcomes such as increased risk of hospitalisation [2].

Research has shown that the type of comorbidity impacts hospital admissions [13] and patient QoL [32]. The present study identified different patterns of comorbidities as CKD progressed. Our results showed a greater prevalence of cardiometabolic comorbidities, such as diabetes and heart problems, in those with more advanced CKD. Specifically, heart problems increased by almost threefold from stages 1–2 to 4–5. Previous studies have observed similar results reporting an increase in cardiovascular comorbidities and a greater risk of cardiovascular events in advanced CKD [20, 21]. Similar to our results, a strong association between CKD and diabetes has previously been reported [5, 19]. Hong et al. [32] observed poor QoL for those with advanced CKD and diabetes, and evidence suggests that those with CKD and multiple cardiometabolic conditions are at heightened risk of hospitalisation [13] and bring greater cost to the healthcare service [5]. The increase in cardiometabolic conditions with disease progression may have important implications for medicine management, hospital admissions, and healthcare costs.

Compared to the more advanced CKD stages, a greater prevalence of lung conditions and mental health disorders were found in stages 1–2. In contrast, Tanaka et al. [21] identified a much higher prevalence of cardiometabolic comorbidities compared to respiratory disorders in both early and advanced stages and Tonelli et al. [2] observed chronic pulmonary disease as one of the five most common comorbidities in stages 3–5. Nonetheless, Tonelli et al. [2] suggested that pulmonary conditions are associated with adverse outcomes in people with CKD, and as such, those living with early CKD are at high risk of hospitalisation. Depression and anxiety are common in those with CKD [11, 12, 33]. Depressive symptoms have been linked to progression to end-stage kidney disease [34] and increased symptoms of anxiety are associated with increased risk of developing comorbidities [12]. As a result, the greater prevalence of mental health disorders observed in stages 1–2 cohort in the present study is worrying and healthcare providers should be vigilant for symptoms that may indicate mental health conditions in early CKD.

We identified diabetes and hypertension as a common comorbidity pairing in stages 3 and 4–5 (> 22% of participants) and these comorbidities formed a cluster in stage 3. To our knowledge, no previous studies have examined comorbidity clusters across the spectrum of CKD; however, diabetes and hypertension have previously been reported as a common comorbidity pair in those living with advanced CKD [20]. This reflects well-established pathophysiological associations [35]. Our results showed that, for stages 1–2, MSK and mental health disorders were a common comorbidity pair, and they also formed a cluster in this stage. This is perhaps owing to their equally high prevalence, although studies examining multimorbidity patterns in a non-CKD population have also demonstrated an association between MSK disorders and mental health problems [31]. We observed a consistent comorbidity clustering between MSK disorders and lung conditions, which occurred three times. In healthy 40–69 year olds, Zemedikan et al. [19] reported a clustering of cardiovascular, MSK, respiratory, and neurodegenerative diseases, and they found that those with COPD are over three times more likely to have osteoporosis. There is also strong evidence to suggest that MSK disorders and COPD coexist together [36].

We found that while the KTx group had a lower prevalence of multimorbidity and fewer comorbidities, they presented with a high burden of comorbidities akin to that seen in stages 1–2. While most studies have examined comorbidities prior to transplantation and their association with post-operative survival [10, 37], our study is the first to explore multimorbidity burden following transplantation. Our results suggest that post-transplant, people may present with comorbidities that resemble patterns of advanced CKD, but they have a lower comorbidity burden.

Our study is strengthened by a large sample of people across the CKD spectrum in the UK, including those with early stages and those with a transplant. Nonetheless, our analysis was limited by the large proportion of White British participants and LTCs attained primarily using self-report. While comorbidities were cross-referenced where possible with clinical data and participant medical records, inadequate recall, lack of awareness around different LTCs, and a lower health literacy in a CKD population [38] may have contributed to misreporting. Participants may have also reported short-term ailments as the study did not record the length of time that comorbidities had existed. However, self-report remains a pragmatic and well-established method for the measurement of multimorbidity [39] and is widely used in this form of research [1, 7, 18, 40]. Previous research has shown moderate to good agreement between self-reported data and administrative data for chronic diseases, including hypertension, diabetes, and myocardial infarction [41, 42]. The list of comorbidities was not exhaustive, and this study would have benefited from a larger sample for stages 1–2. As shown in people with type 2 diabetes [43, 44], an expert-driven approach to stratification of CKD participants in this study could more clearly indicate mortality patterns and the healthcare resources required to support each patient group. Alternative multimorbidity patterns may have been identified had additional MLTCs and a larger early CKD population been analysed. Crudely taken, the prevalence of many common comorbidities was similar to those identified in other studies using alternate means (e.g., disease index read codes). The use of frequency ‘counts’ meant that no weighting of diseases regarding severity or prognosis was possible [40].

## Conclusion

In people with CKD, multimorbidity adds risk of hospitalisation and complexity to treatment medication regimes, and self-management strategies. Our results showed a high level of multimorbidity across the spectrum of CKD, including those with early stages and those with a transplant. We identified different patterns of comorbidities across the different stages. Practice guidelines are often ‘disease-centred’ and do not consider the potential for competing therapeutic objectives, drug interactions, or life expectancy. Therefore, healthcare professionals should adopt a ‘person-centred’ approach, as people with CKD may require different integrated care and management strategies depending on their disease progression. Our findings may help to prevent and manage multimorbidity as people progress from early CKD to advanced CKD.

## Supplementary Information

Below is the link to the electronic supplementary material.

Supplementary file1 (DOCX 51 KB)

## Data Availability

The data that support the findings of this study are available from the corresponding author upon reasonable request.

## Abbreviations

ACR: Albumin to creatinine ratio
BMI: Body mass index
BP: Blood pressure
CKD: Chronic kidney disease
CHF: Chronic heart failure
COPD: Chronic obstructive pulmonary disease
eGFR: Estimated glomerular filtration rate
Hb: Haemoglobin
HTN: Hypertension
IQR: Interquartile range
KTx: Kidney transplant
LTC: Long-term condition
MH: Mental health
MLTC: Multiple long-term conditions
MM: Multimorbidity
MSK: Musculoskeletal
NICE: National institute for health and care excellence
PCA: Principal component analyses
QoL: Quality of life
SD: Standard deviation
