# Supplementary material for: Multimorbidity prevalence and patterns in chronic kidney disease: findings from an observational multicentre UK cohort study
Source: Int Urol Nephrol. 2023 Feb 21;55(8):2047–57. doi: 10.1007/s11255-023-03516-1 (PMC10329585; doi:10.1007/s11255-023-03516-1)
Supplement: Supplementary file 1 — Supplementary file1 (DOCX 51 KB) [file 11255_2023_3516_MOESM1_ESM.docx]

**Supplementary Material**

Supplementary Figure S1. STROBE flow diagram

Supplementary Table S1: Characteristics of multimorbid participants vs non-multimorbid participants

Supplementary Table S2: Predictors of multimorbidity for all stages of CKD

Supplementary Table S3: Prevalence of each comorbidity across all stages

Supplementary Table S4: Cluster factor loadings for each stage following principal component analysis

Supplementary Table S5: Comorbidity clusters for all stages of CKD

**Supplementary Figure S1. STROBE flow diagram**


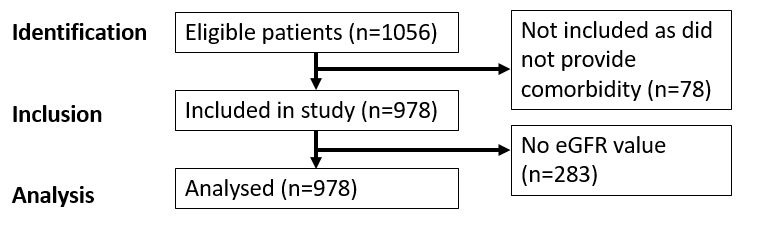


**Notes:** n=283 did not have eGFR value but were included in all analysis of total population

**Abbreviations:** eGFR: estimated glomerular filtration rate

**Supplementary Table S1: Characteristics of multimorbid participants vs non-multimorbid participants**

|  | CKD only (n=42) | Multimorbid (n=936) | P value |
| --- | --- | --- | --- |
| Age (years) | 57.1 ± 16.1 | 66.7 ± 13.8 | <0.001 |
| Male, n (%) | 23 (54.8) | 564 (60.4) | 0.520 |
| White British | 38 (92.3) | 860 (92.9) | 0.771 |
| South Asian | 2 (4.9) | 21 (2.3) | 0.259 |
| Other white | 1 (2.4) | 28 (3.0) | 1.000 |
| Other | 0 | 17 (1.8) | 1.000 |

**Abbreviations:** CKD: chronic kidney disease.

**Supplementary Table S2: Predictors of multimorbidity for all stages of CKD**

| **Parameter Estimates^a^** | | | | | | | | |
| --- | --- | --- | --- | --- | --- | --- | --- | --- |
|  | | Estimate | Std. Error | Wald | df | Sig. | 95% Confidence Interval | |
|  |  |  |  |  |  |  | Lower Bound | Upper Bound |
| **Stage 1-2** | | | | | | | | |
| Location | Age | -.025 | .015 | 2.763 | 1 | .096 | -.054 | .004 |
|  | [Sex=1] | .524 | .490 | 1.148 | 1 | .284 | -.435 | 1.484 |
|  | [Sex=2] | 0^b^ | . | . | 0 | . | . | . |
|  | **Stage 3a&b** | | | | | | | |
| Location | Age | -.038 | .011 | 11.249 | 1 | <.001 | -.060 | -.016 |
|  | [Sex=1] | .039 | .277 | .020 | 1 | .888 | -.505 | .583 |
|  | [Sex=2] | 0^b^ | . | . | 0 | . | . | . |
|  | **Stage 4-5** | | | | | | | |
| Location | Age | -.019 | .008 | 5.921 | 1 | .015 | -.035 | -.004 |
|  | [Sex=1] | .396 | .227 | 3.055 | 1 | .080 | -.048 | .840 |
|  | [Sex=2] | 0^b^ | . | . | 0 | . | . | . |
|  | **KTx** | | | | | | | |
| \| Location \| Age \| -.019 \| .008 \| 5.921 \| 1 \| .015 \| -.035 \| -.004 \| \| --- \| --- \| --- \| --- \| --- \| --- \| --- \| --- \| --- \| \| [Sex=1] \| .396 \| .227 \| 3.055 \| 1 \| .080 \| -.048 \| .840 \| \| [Sex=2] \| 0^b^ \| . \| . \| 0 \| . \| . \| . \| | Age | -.032 | .011 | 8.740 | 1 | .003 | -.053 | -.011 |
|  | [Sex=1] | .071 | .274 | .068 | 1 | .795 | -.465 | .607 |
|  | [Sex=2] | 0^b^ | . | . | 0 | . | . | . |

**Abbreviations:** KTx: Kidney transplant

**Supplementary Table S3: Prevalence of each comorbidity across all stages**

|  | **All CKD** (n=978) | **Stage 1-2**  (n=56) | **Stage 3a&b** (n=185) | **Stage 4-5** (n=278) | **KTx**  (n=176) | **P value^1^** | **P value^2^** |
| --- | --- | --- | --- | --- | --- | --- | --- |
| Hypertension | 492 (50.3) | 31 (55.3) | 117 (62.9) | 173 (62.2) | 107 (60.8) | 0.556 | 0.821 |
| MSK disorders | 447 (45.7) | 31 (55.3) | 78 (42.2) | 130 (46.8) | 72 (40.9) | 0.113 | 0.244 |
| Heart problems | 350 (35.8) | 8 (14.3) | 65 (35.1) | 112 (40.3) | 46 (26.1) | 0.013 | 0.019 |
| Diabetes | 325 (33.2) | 12 (21.4) | 59 (31.9) | 98 (35.3) | 46 (26.1) | 0.431 | 0.478 |
| Circulatory disorders | 268 (27.4) | 17 (30.4) | 53 (28.6) | 85 (30.6) | 39 (22.2) | 0.841 | 0.507 |
| Lung conditions | 266 (27.2) | 19 (33.9) | 42 (22.7) | 83 (29.9) | 35 (19.9) | 0.106 | 0.092 |
| Obesity | 265 (27.1) | 14 (25.0) | 52 (28.1) | 71 (25.5) | 35 (19.9) | 0.709 | 0.182 |
| Mental health disorders | 200 (20.4) | 17 (30.4) | 38 (20.5) | 53 (19.1) | 31 (17.6) | 0.845 | 0.289 |
| Cancer | 186 (19.0) | 5 (8.9) | 30 (16.2) | 60 (21.6) | 28 (15.9) | 0.342 | 0.501 |
| Liver conditions | 81 (8.3) | 4 (7.1) | 17 (9.2) | 19 (6.8) | 14 (7.9) | 0.581 | 0.784 |
| Stroke | 86 (8.8) | 3 (5.4) | 13 (7.0) | 21 (7.6) | 14 (7.9) | 0.957 | 0.830 |

**Abbreviations:** CKD: chronic kidney disease; KTx: Kidney transplant; MSK: musculoskeletal disorders.

**Supplementary Table S4: Cluster factor loadings for each stage following principal component analysis**

|  | **Cluster 1** | **Cluster 2** | **Cluster 3** | **Cluster 4** |
| --- | --- | --- | --- | --- |
| **Stage 1-2** | | | | |
| Circulatory | .690 |  |  |  |
| Obesity | .687 |  |  |  |
| Heart problems | .618 |  |  |  |
| Hypertension | -.512 |  |  |  |
| Diabetes |  | .762 |  |  |
| Lung conditions |  | .682 |  |  |
| MSK |  |  | .629 |  |
| MH |  |  | .595 |  |
| Liver conditions |  |  |  | .801 |
| Stroke |  |  |  | .694 |
| Cancer |  |  |  |  |
| **Stage 3a and b** | | | | |
| Liver conditions | .733 |  |  |  |
| Stroke | .685 |  |  |  |
| Cancer |  |  |  |  |
| MH |  |  |  |  |
| Lung conditions |  | .755 |  |  |
| MSK |  | .671 |  |  |
| Obesity |  |  | .790 |  |
| Circulatory |  |  | .542 |  |
| Diabetes |  |  |  | .814 |
| Hypertension |  |  |  | .700 |
| Heart problems |  |  |  |  |
| **Stage 4-5** | | | | |
| MSK | .652 |  |  |  |
| Lung conditions | .649 |  |  |  |
| Liver conditions | .555 |  |  |  |
| Diabetes |  | -.684 |  |  |
| Obesity |  | .649 |  |  |
| Circulatory |  |  |  |  |
| Hypertension |  |  | .757 |  |
| Stroke |  |  | .611 |  |
| Heart problems |  |  |  | .839 |
| MH |  |  |  | -.530 |
| Cancer |  |  |  |  |
| **KTx** | | | | |
| Lung conditions | .727 |  |  |  |
| MSK | .693 |  |  |  |
| Cancer |  | .799 |  |  |
| Diabetes |  | .738 |  |  |
| Hypertension |  |  | -.769 |  |
| Circulatory |  |  | .663 |  |
| Liver conditions |  |  |  | -.731 |
| MH |  |  |  | .684 |
| Heart problems |  |  |  |  |
| Stroke |  |  |  |  |
| Obesity |  |  |  |  |

**Abbreviations:** HTN: hypertension; KTx: Kidney transplant; MH: mental health disorders; MSK: musculoskeletal disorders.

**Supplementary Table S5:** **Comorbidity clusters for all stages of CKD**

|  | **Cluster 1** | **Cluster 2** | **Cluster 3** | **Cluster 4** |
| --- | --- | --- | --- | --- |
| **Stage 1-2** | *Circulatory, obesity, heart problems, HTN* | *Diabetes, Lung* | *MSK, MH* | *Liver, stroke* |
| **Stage 3a&b** | *Stroke, Liver* | *Lung, MSK* | *Obesity, Circulatory* | *Diabetes, HTN* |
| **Stage 4-5** | *MSK, lung, liver* | *Diabetes, Obesity* | *HTN, stroke* | *Heart problems, MH* |
| **KTx** | *Lung, MSK* | *Cancer, Diabetes* | *HTN, circulatory* | *Liver, MH* |

**Abbreviations:** HTN: hypertension; KTx: Kidney transplant; MH: mental health disorders; MSK: musculoskeletal disorders.
